# Branching Shoots and Spikes from Lateral Meristems in Bread Wheat

**DOI:** 10.1371/journal.pone.0151656

**Published:** 2016-03-17

**Authors:** Ying Wang, Fang Miao, Liuling Yan

**Affiliations:** Department of Plant and Soil Sciences, Oklahoma State University, Stillwater, Oklahoma, 74078, United States of America; Institute of Genetics and Developmental Biology, CHINA

## Abstract

Wheat grain yield consists of three components: spikes per plant, grains per spike (*i*.*e*. head or ear), and grain weight; and the grains per spike can be dissected into two subcomponents: spikelets per spike and grains per spikelet. An increase in any of these components will directly contribute to grain yield. Wheat morphology biology tells that a wheat plant has no lateral meristem that forms any branching shoot or spike. In this study, we report two novel shoot and spike traits that were produced from lateral meristems in bread wheat. One is supernumerary shoot that was developed from an axillary bud at the axil of leaves on the elongated internodes of the main stem. The other is supernumerary spike that was generated from a spikelet meristem on a spike. In addition, supernumerary spikelets were generated on the same rachis node of the spike in the plant that had supernumerary shoot and spikes. All of these supernumerary shoots/spikes/spikelets found in the super wheat plants produced normal fertility and seeds, displaying huge yield potential in bread wheat.

## Introduction

Bread wheat, *Triticum aestivum* (2n = 6X = 42, AABBDD), displays various morphological traits that are produced due to the number and constitution of chromosomes during the evolution and natural selection, providing an exceptionally rich genetic resource for wheat improvement [1–3]. However, regardless of chromosome numbers, wheat grain yield of a typical wheat cultivar consists of three components: spikes per plant, grains per spike (*i*.*e*. head or ear), and grain weight. The grains per spike can be dissected into two subcomponents: spikelets per spike and grains per spikelet. An increase in any of these components will directly contribute to grain yield [4].

One spike is produced on the top of the main stem and each of the tillers that are fertile in a normal wheat cultivar, and those tillers that do not produce spike are referred to sterile tillers [5, 6]. The main stem and the tillers of a plant reside on the same compact and unelongated basal internodes under the ground (joined internodes) [7]. No tiller is developed from an axillary bud at the axil of a leaf on the elongated internode above the ground, and those axillary buds on aerial leaves do not develop but die due to hypoplasia [4, 8]. In other plant species, a tassel in maize or a panicle in rice can be produced directly from an axillary bud at the leaf axil on the elongated aboveground internodes; therefore, and one stem carries multiple tassels or panicles. If a tiller is produced from an axillary bud at the leaf axil on the elongated aboveground internodes, then branching shoots and multiple tassels or panicles can be developed in the same shoot [9, 10].

A spike in a normal wheat cultivar produces approximate 15–25 spikelets, each residing on per rachis node of the spike of the main stem or the fertile tillers [11]. The reproductive spike develops from an apex meristem to a spikelet meristem as a terminal meristem to form the final floral organs. If the spikelet meristem develops as a lateral spike instead of a spikelet, however, a branching spike is produced in the same spike.

A spikelet in a normal wheat spike produces 5–10 florets encompassed by two small bract leaves [12]. The reproductive spikelet develops as a terminal meristem on the spike and later differentiates into the floral meristem. If the floral meristem develops as a lateral spikelet instead of a floret, however, a branching spikelet is produced. The branching spike in wheat was discovered nearly as early as one century ago (Percival, 1921 cited in Nature by Shaman 1944) [13], and this trait was reported in tetraploid species, including ‘*Miracle*’ wheat in *T*. *turgidum* and other branched wheat in *T*. *dicoccum*, *T*. *polonicum*, *T*. *dicoccum*, and *T*. *vulgare* [14]. Branching spikelets can be classified into three types, twin spikelets situating on the same rachis node vertically or horizontally [15], triple spikelets residing on one spike rachis node in a similar pattern to six-rowed barley [16], and multiple spikelets generated on one node of spike rachis, which is referred to as a “multirow spike” (MRS) in wheat, in a similar pattern to a “monstrosum ear” in rye [17]. There is some confusion about supernumerary spikelet types, due to the presence of “hetero-branching” types. Other terms such as ‘short ramified spikelets’, ‘long ramified spikelets’ [18], and four row spikelets [19] are also used to distinguish supernumerary spikelet types.

In this study, we report that hexaploid bread wheat plants were able to produce not only supernumerary shoot developed from an axillary bud at the axil of leaves on the elongated internodes of the main stem but also supernumerary spike that was generated from a spikelet meristem on a spike. In addition, supplementary spikelets were observed on the same rachis node of the spike in the plant that had supernumerary shoot and spikes. The super wheat plant having the **s**upernumerary **s**hoots/**s**pikes/**s**pikelets is referred as to ‘4S wheat’.

## Materials and Methods

A population of 154 BC_1_F_2_ progeny plants was generated from crosses between two winter wheat cultivars with distant genetic backgrounds, ‘2174’ that is a hard red winter wheat and ‘XJ2012’ that is a Chinese landrace dwarf lines and extensively utilized in breeding programs. The XJ2012/2174/2174 progeny plants were created to introduce potential genes for spikes and other agronomic traits in XJ2012 into the locally adapted cultivar 2174.

The population and parental lines were grown in a greenhouse with constant temperature (20–25°C) and long day condition (16 hour/8 hour for light/dark). The plants were not vernalized throughout the experiment. The plants were grown in pots with a commercial soil (BWI, TX). A higher level of nitrogen fertilizer (100 mg N /kg soil) was provided to a cone in which only one plant was tested, and tap water was utilized throughout the growth season.

The transcription factor gene *TB1*, was reported to repress axillary bud outgrowth in rice [20]. The wheat orthologues of the gene rice *OsTE1* were isolated by PCRs, using primers TaTB-AF1 (5-TTCCACCCGCAGACACACAG-3) and TaTB-AR1 (5-GTGTACGTGCAAGGCGAGACAGT-3) for homoeologous *TaTB-A1* on chromosome A. The PCRs were performed using standard protocols for LongAmp *Taq* DNA polymerase (New England BioLabs) and 35 thermal cycles after denature at 95°C for 5 minutes, each cycle consisting of 94°C for 30 sec, 55°C for 30 sec and 72°C for 1 min. *TE1* was reported to control the formation of the shoot branches by its direct repression on the axillary buds on the elongated internodes in rice [21]. The wheat orthologues of the gene rice *OsTE1* were isolated by PCRs, using primers primers TaTE-DF1 (5- GACGCCATCAACAGCAAGCGG-3) and TaTE-DR1 (5- CATGTTTCCCTGACATGAACATTTCCCAA-3) for homoeologous *TaTB-D1* on chromosome D. The PCRs were performed using the same cycling program as used for *TB1* 35 except for extension time that was extended to 4 mins at 72°C for each cycle.

The shortening stems/shoots of the 4S wheat were observed under microscopy. Histological methods were used to check how supernumerary shoot and spikes were produced. Spikelets per spike and grains per spike were counted. The 1000-grain weight were determined and calculated to grain weight with 13% standard moisture content.

## Results

### Discovery of novel spike traits

The parental lines, F_1_ plants, and BC_1_F_1_ plants generated from crosses between 2174 and XJ2012 showed normal development in spike and other agronomic traits when the plants were continuously grown in a greenhouse with constant temperature and long day condition. However, 13 plants in the population of 154 BC_1_F_2_ XJ2012/2174/2174 lines were observed to have novel traits.

**Fig 1:**
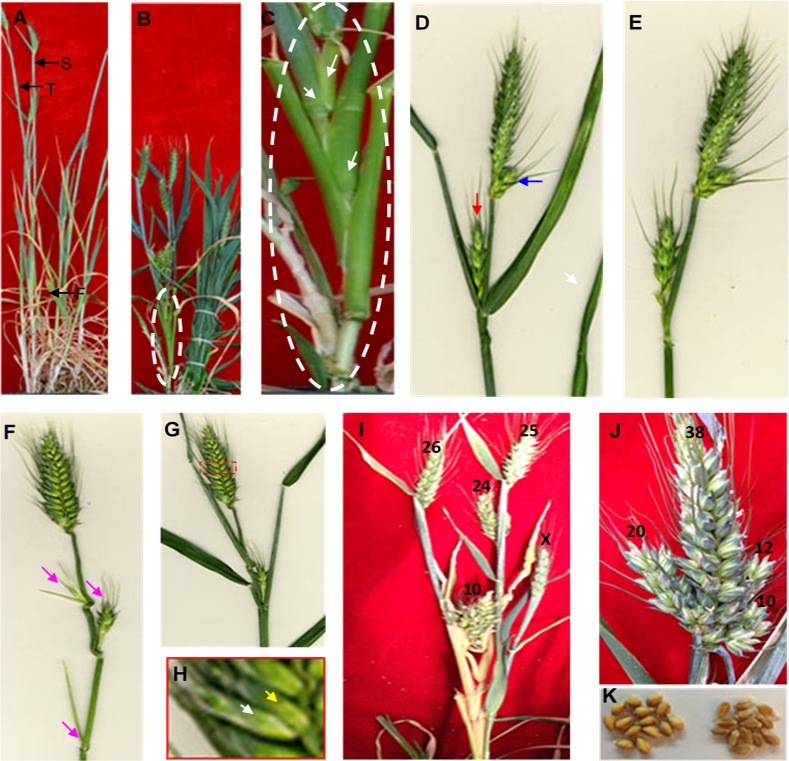
Morphological characteristics of the 4S wheat plant. **A.** A normal plant with one spike on one stem. **B.** A 4S wheat plant. The part to be featured is indicated with a white line. **C.** Tillers from the axillary buds at the axil of each of three aerial leaves on the elongated internodes. **D.** An axillary bud at the axil of the flag leaf on the elongated internodes developed directly to a spike is indicated by an arrow in red, and a spikelet meristem advanced to a spike is indicated by an arrow in blue. **E.** The spike developed from the axil of the flag leaf on the elongated internodes in Fig 1D is exposed after the flag leaf is removed. **F.** The developing spikes or tillers from the axils of three leaves on the elongated internodes are exposed and indicated by arrows in purple after the aerial leaves are removed. **G.** Supernumerary spikelets developed from on rachis node are squared in red line. **H.** The two spikelets on the same rachis node are featured to show that the normal spikelet indicated by a white arrow and the supernumerary spikelet indicated by a yellow arrow reside on the same rachis node. **I.** A stem with branching shoot. The numbers indicate grains per spike. **J.** A spike with branching spikes with normal fertility. The numbers indicate grains per spike. **K.** The seeds from the supernumerary spikes are normal (on the left), and the 2174 seed are shown on the right.

As shown in Fig 1A, a normal adult plant of hexaploid wheat bore several tillers. The tillers developed in a relatively simple pattern, a primary tiller from an axillary bud of the main stem, and a secondary tiller from an axillary bud of the primary tiller, and so on, but all of the tillers in the plant were developed from axillary buds of the leaves from the nodes of unelongated basal internodes that were compacted or joined and resided under the ground. No tiller was developed from an axillary bud at the axil of a leaf on the elongated internodes above the ground. The developed tillers either became infertile to die due to hypoplasia or advanced to infertile tillers. One spike was developed on the top of the main stem or each of the fertile tillers. The developmental pattern of tillers and spikes is a common feature of temperate crops including wheat, barley, and rye.

The developmental pattern of tillers and spikes, however, was changed in the 4S wheat, and multiples shoots and multiple spikes on the main stem and a fertile tiller (Fig 1B). Tillers, as indicated by arrows in Fig 1C, were developed from axillary buds at the axils of leaves on the elongated internodes of the main stem, producing branching tillers from the elongated internodes above the ground. These branching tillers were fertile; therefore, the main stem bore four heads, one on the main stem and three on the fertile tillers, thereby forming supernumerary shoots.

In the 4S wheat, not only the axillary buds at the axils of leaves on the elongated internodes developed to tillers, but also the axillary bud on the axil of the flag leaf advanced to a spike directly (Fig 1D). After the flag leaf of a tiller was excised, the spike was exposed to show that the spike was developed from the lateral meristem, thereby forming supernumerary spikes (Fig 1E). The axillary bud at the axil of each aerial leaf on the elongated internodes of the tiller developed as a lateral organ, which either produced a spike or died due to hypoplasia (Fig 1F).

The lateral meristem cells in the rachis node of spike in the 4S wheat were developed to not only spikelets but also spikes. As shown in Fig 1E and Fig 1F, a spikelet meristem on the basal node of a spike advanced to a spike instead of a spikelet, thereby producing a branching spike. Normal spikelets were produced on other nodes of the same spike rachis. Three spikes were produced on the same rachis of a spike, also forming supernumerary spikes (Fig 1J).

Supernumerary spikelets were also observed to develop from on the rachis node of the spike in the 4S wheat. Two spikelets on the same rachis node are featured to show that the normal spikelet, indicated by a white arrow, and the supernumerary spikelet, indicated by a yellow arrow, resides on the same rachis node (Fig 1G and 1H), thereby forming supernumerary spikelets.

All of these supernumerary shoots/spikes/spikelets found in the wheat plants had normal fertility and grains (Fig 1K). A single stem possessing four branching shoots totally produced as many as 85 grains (Fig 1I), and a single spike possessing four branching spikes totally produced as many as 80 grains (Fig 1J). When grown in the same small cone, a normal wheat plant produced 10–20 grains per shoot or per spike.

It is noteworthy that accompanying traits in the 4S wheat plants showed delayed flowering time, dark green leaves, and reduced plant height, in addition to multiple tillers (Fig 1B). These accompanying traits with the 4S wheat were also observed in rice that has a mutated *te* gene (21).

### Histological and molecular analyses of the 4S wheat traits

**Fig 2:**
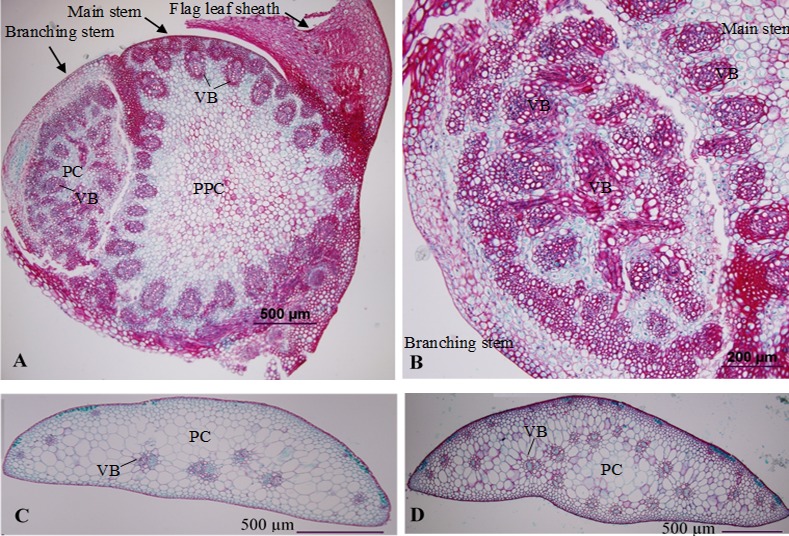
Histological sections of the 4S wheat. **A.** The lateral meristem of the axillary bud at the axil of the flag leaf. **B.** Featuring the branding stem from the axillary bud at the axil of the flag leaf shown in Fig 2A. **C.** The spike axis of the 4S wheat. **D.** The spike axis of 2174 wheat. VB: vascular bundle; PPC: pith parenchymal cell; PC: parenchymal cell.

Histological analysis showed that the spike from the axillary bud at the axil of the flag leaf was developed from the lateral meristem cells (Fig 2A). Sections on featured the lateral meristems revealed that minor veins and irregular vascular patterns were generated in the axillary bud that developed to a spike (Fig 2B). Sections on the spike axis of the main stem revealed that the 4S wheat had less vascular patterns in the spike axis bearing supernumerary spikes (Fig 2C) compared with cultivar 2174 (Fig 2D).

However, no deletion or functional mutations was observed in the *TaTB-A1* gene between the two parental lines. *TaTE1* is a gene that consists of 10 exons and 9 introns and encodes 513 amino acids in wheat. The orthologous *TaTE-D1* gene in hexaploid wheat corresponded to sequences in contig 14307730 from IWGSC_chr4DL. The 3,933 bp fragment including 84 bp in upstream from the start codon and 254 bp from the stop codon and 3,595 for the complete gene region were sequenced, but no deletion or functional mutations in this gene between the two parental lines.

The axillary bud outgrowth in rice was related to *TB1* and *TE1* [20, 21]. The *TB1* and *TE1* sequences of the wheat orthologues were identified. *TB1* is a gene that has a single exon encoding 352 amino acids in wheat, and the orthologous *TaTB-A1* genes in hexaploid wheat corresponded to sequences in contig 7013613 from IWGSC_chr4AL. However, no deletion or functional mutations was observed in the *TaTB-A1* gene between the two parental lines. *TaTE1* is a gene that consists of 10 exons and 9 introns and encodes 513 amino acids in wheat. The orthologous *TaTE-D1* gene in hexaploid wheat corresponded to sequences in contig 14307730 from IWGSC_chr4DL. The 3,933 bp fragment including 84 bp in upstream from the start codon and 254 bp from the stop codon and 3,595 for the complete gene region were sequenced, but no deletion or functional mutations in this gene between the two parental lines.

The segregation of the 4S wheat plants and normal plants also fits to a genetic model. The chi-square test showed that the segregation ratio was consistent to 15:1 (X^2^ = 0.3006, *df* = 1, *p* = 0.583), suggesting that the 4S wheat traits could be governed by two independent recessive genes in the BC_1_F_2_ genetic backgrounds. A 3:1 segregation ratio of the normal traits and the 4S traits was expected to observe in ¼ of BC_1_F_3_ populations generated from individual BC_1_F_2_ plants. However, when BC_1_F_3_ populations generated from individual BC_1_F_2_ plants were tested, no similar traits were observed.

## Discussion

The 4S wheat plants showed capacity to produce axillary branch meristems for additional or supernumerary shoots, spikes, as well as spikelets. More than one spikelet developed as an axillary meristem from the same rachis node, hence, the spikelet node bore supernumerary spikelets. The 4S wheat traits were unexpectedly observed from the progeny plants of the backcrosses between two common wheat cultivars with distant genetic backgrounds. To our knowledge, there is no report that a lateral shoot or spike is formed on elongated internodes in temperate cereal species, so the traits are novel.

The population was tested in a photoperiod-temperature controlled greenhouse; therefore, the 4S wheat traits were not resulted from abiotic stresses. However, the expected trait was not observed in the BC_1_F_3_ populations. It is less likely that the 4S wheat traits were caused by any interaction between genetic factors and environmental cues. It is more likely that the genetic factors from the dwarf wheat resulting in the 4S traits have been masked in BC_1_F_3_ plants or lost during self-pollination. Although it is not known about mechanisms underlying the novel traits, the 4S plants have demonstrated that wheat has potential in branching shoots and branding spikes.

From the view of plant biology, a wheat tiller is produced from an axillary bud at the axil of leaves on the unelongated internodes but not on elongated internodes, and those axillary buds on aerial leaves do not develop [7, 8]. However, we have demonstrated that axillary buds at the axil of leaves on the elongated internodes are viable and can develop into spikes or fertile tillers. This study has advanced our understanding and knowledge of the genes and genetic pathways underlying spike and spikelet development in wheat and of the genome biology in plant architecture.

It was reported that supernumerary spikelet in wheat is controlled by one or two recessive genes [16, 22–26]. It was also reported that the trait for four-rowed spike and ramified spike is associated with a major recessive gene on chromosome 2A and numerous minor genes including one on chromosome 2B [18–19], and the ‘triple-spikelet’ trait in a Tibetan landrace of bread wheat is dominantly determined by a major gene on chromosome 2A [27]. Strong inhibitors of supernumerary spikelets may be located on chromosomes 2DS and 2AL in Chinese Spring [15, 19, 28]. However, no gene for spike architecture in wheat has been cloned, except the wheat *Q* gene that is related to spike shape and the variation in the free-threshing character and many other domestication-related traits such as glume toughness, rachis fragility, and spike length [29, 30].

A barley spike is an indeterminate type that has three spikelets on each rachis node including one central and two lateral spikelets. According to lateral spikelet fertility, barley is classified into two types: two-rowed barley in which the central spikelet is fertile and produces grains but the two lateral spikelets remain sterile, and six-rowed barley in which all three spikelets are fertile and develop into grains [31]. Three genes, *six-rowed spike1* (*vrs1*), *vrs4*, and *Intermedium-C* (*Int-c*) responsible for the rowed spikelet phenotype, have been cloned. *Vrs1*, which encodes a homeodomain-leucine zipper class I transcription factor, is a negative regulator of lateral spikelet fertility [31]. *Int-c*, which is an ortholog of the maize domestication gene *TB1*, modifies lateral spikelet development [32]. *Vrs4*, which is an ortholog of the maize transcription factor *Ramosa 2*, regulates lateral spikelet fertility and indeterminate triple spikelet meristems, thereby producing additional spikelets/florets [33]. However, the genes for the spike characters unique to barley may not be related to the 4S wheat traits.

The 4S wheat plant has branching spikes, the shape of which looks like a male inflorescence in maize. The 4S wheat plant produced multiple shoots with fertile heads on one stem, the shape of which is similar to a rice ‘*te*’ mutant. The comparative traits in the crops suggest that the two distant species may have common genes controlling spike morphology. The number of fertile tillers bearing multiple spikes is an important trait for the ideal plant types in crops [27]. Several key regulators involved in axillary meristem formation have been identified in plants, including *REVOLUTA* [34], *LAS* [35] and *RAX1* [36] in Arabidopsis, and *BA1* in maize [37]. In rice, tillering is controlled by *MOC1* [38] and *LAX* genes [39], and the loss-of-function mutations in the *MOC1* and *LAX2* result in a single main culm phenotype. As a master switch of tillering, *MOC1* also promotes tillering by up-regulating the expression of the transcription factor gene *TB1*, which represses axillary bud outgrowth [39]. *TB1* antagonizes the activity of *MADS57*, which represses D14 (dwarf 14), a negative regulator of tilling [40]. Strigolactone inhibits axillary bud growth, thereby negatively regulating tillering. Mutations in genes involved in strigolactone biosynthesis and signaling cause the increased tiller number and dwarf phenotype, and these mutants in rice are mainly designated as dwarf (d) mutants [41]. These genes function in the same pathway controlling the tillers that develop from axillary buds at the axils of leaves on the unelongated basal internodes. *TE1* [21], which is a rice homologue of *Cdh1*, controls the formation of the shoot branches by its direct repression on the axillary buds on the elongated internodes or its mediation of the degradation of MOC1 protein. Although the mechanisms controlling the lateral branching shoots in wheat are largely unknown, the sequences of the genes in the cereal crops can be used to characterize the genes controlling the 4S trait in wheat.

Great efforts have been made to incorporate branching *T*. *turgidum* spike type into common wheat by using wide crosses between *T*. *aestivum* L. and branching forms of *T*. *turgidum* L. Although various spike types are observed, such as a compact spike in *T*. *compactum* (club wheat) or a lax spike in spelt wheats (*T*. *aestivum* ssp. *spelta*), branching spike is rarely detected in hexaploid wheat. More than 13 branching forms of spikes have been produced in the same populations by several backcrosses between tetraploid and hexaploid wheat [42, 43], but all of them are sterile or of low fertility, limiting utilization of various forms of spikes.

The 4S wheat plants have shown accompanying traits including multiple tillers, dark green leaves, and reduced plant height, but apparently none of these phenotypes could be a negative factor for grain yield. In crops, reduced plant height is especially important for lodging-resistance in securing potential grain production. The Green Revolution in 1960s was marked by the use of semi-dwarf and lodging-resistant varieties of rice and wheat through manipulating biosynthesis and signaling of gibberellin (GA), a phytohormone controlling cell elongation in plants. Although a little information is available for genes responsible for branching meristems in the 4S wheat, the never-seen but interesting and valuable traits in the ‘4S’ wheat showed huge potential for wheat grain yield.
